# Guidelines for Developing Yoga Interventions for Randomized Trials

**DOI:** 10.1155/2012/143271

**Published:** 2012-10-02

**Authors:** Karen J. Sherman

**Affiliations:** ^1^Group Health Research Institute, Group Health Cooperative, 1730 Minor Avenue, Suite 1600, Seattle, WA 98101, USA; ^2^Department of Epidemiology, University of Washington, Seattle, WA 98195, USA

## Abstract

Little guidance is available to assist researchers in developing treatment protocols for research on yoga for health concerns. Because yoga is a complex multifactorial mind-body discipline historically developed for nonmedical purposes, numerous decisions must be made in order to thoughtfully develop such protocols. In this paper, a systematic approach is proposed to assist researchers in selecting an intervention that is appropriate for the condition under consideration and explicitly developed. Researchers need to consider the type or “style” of yoga, the components to include (e.g., breathing exercises, postures) as well as the specific protocol for each component, the dose to be delivered (frequency, duration of practice, and the total duration of practice), and issues related to selection of instructors and monitoring the fidelity to the intervention. Each of these domains and the key issues for the development of protocols is discussed. Finally, some areas for further research related to protocol development are recommended.

## 1. Introduction

One of the most unexplored aspects of designing clinical studies of complementary and alternative (CAM) therapies is the thoughtful, systematic development of treatment protocols. Such protocols can be challenging to develop because of the tension between the desire for reproducible protocols in a scientific setting and the fluid and adaptable nature of CAM therapies such as yoga, wherein a plethora of treatment elements and variations exist and adaptation to the individual is common. Yet, protocols are critical because they comprise the intervention that is tested in a clinical trial. Every effort should be made to ensure that such protocols are well designed, suitable for the population under study, and would be recognized by other yoga experts as being reasonable treatments for the condition under study.

Even though little guidance assists researchers in developing protocols for most CAM therapies, a striking exception has been acupuncture, where there has been considerable work on developing treatment protocols [1–3] and even an extension of the CONSORT statement [4] for reporting appropriate details of such protocols [5]. From a researcher's perspective, acupuncture has many broad features in common with yoga. These include a multiplicity of “styles,” different elements of treatment, a wide variety of prescriptions for specific conditions, tendency to customize treatments to individuals, and differences between practitioners' training and focus. In this paper, a systematic approach for developing treatment protocols for studies of yoga is adapted and extended from previous work on acupuncture protocols. While the approach is focused on yoga studies conducted in the Western world, it could be appropriately adapted for studies in India and other non-Western countries as well.

## 2. Methods and Results

### 2.1. A Therapeutic Perspective on Selecting Yoga Treatments

The origins of yoga are rooted in antiquity as a spiritual discipline for self-development. Of note, the only yoga posture described in the Yoga Sutras, the first text on yoga, was sitting. Traditionally, even hatha yoga, the physical aspect of yoga practice, was described as a spiritual discipline [6]. However, contemporary research on yoga in the Western world is primarily focused on potential physical and mental health benefits and thus, the discussion about the development of treatment protocols is oriented to this goal. Current clinical research on yoga focuses primarily on efficacy studies of yoga evaluating the therapeutic value under optimal circumstances. These presently serve as “proof-of-concept” studies. A second class of studies, focused on effectiveness (i.e., the therapeutic value of yoga as it is actually practiced in the community), would allow a much broader treatment protocol.

We propose that each of the following domains needs to be addressed when developing an appropriate and robust yoga protocol for efficacy trials.

1. Style of yoga.
2. Dose and delivery of yoga.
3. Components of the yoga intervention.
4. Specific class sequences.
5. Dealing with modifications.
6. Selection of instructors.
7. Facilitation of home practice.
8. Measurement of intervention fidelity over time.

Some of these domains are the clear purview of experts in teaching yoga for therapeutic purposes, but researchers should be aware that they will need to work with such experts to ensure that the proposed intervention meets the needs of rigorous science as well as therapeutic soundness.

While each of these elements is important, specific decisions made in developing a specific protocol will depend on the condition or disease under study, the study population, likely comorbidities, and the availability of suitable instructors.

To help the reader understand the state of discussion regarding protocol development, we have included pertinent information about several of these domains using data from relatively recent clinical trials of yoga for cardiovascular risk factors and disease [7–19], symptoms related to cancer or its treatment [20–31], back pain [32–41],and other types of pain [42–49]. We selected these conditions because they represent a range of reasons for which yoga has been studied and often include a broad range of outcomes that yoga is postulated to influence.

#### 2.1.1. Style of Yoga

Yoga has traditionally been practiced in the context of a lineage, with different variations passed down from teacher to student. In the West particularly, this results in a number of different “styles” of yoga, each of which has slightly different emphases. These vary in physical intensity from gentle to vigorous, in performance of the posture from adapting the pose to fit the body type to precise alignment of the body, in how much they include an explicit emphasis on the “spiritual,” in how long postures might be held, and whether they “flow” from one to another. Some styles focus on special breathing techniques while in the postures. Numerous other differences exist as well. The term “hatha yoga” would refer to the general practice of physically focused yoga, without reference to another style. Whether these stylistic differences are important from a therapeutic perspective, or they are simply various variations on a theme that each has its own internal coherence, is unknown and should be explicitly examined in future studies.

Although yoga lineages from many parts of India are now taught in the West, T Krishnamacharya, an Indian yoga expert from Mysore, had an extraordinary influence on yoga in the West because he trained several well-known hatha yoga teachers including BKS Iyengar and Pattabhi Jois (who developed his style of “Ashtanga Yoga”). More recently, Westerners have been adapting and sometimes blending different yoga lineages to create a variety of new styles.

When selecting a particular style for research on health, a major consideration is the need to use a style that is safe and appropriate for the patient population. Even then, there are likely to be multiple styles that may be appropriate. Several considerations can be used to narrow the choice further. It could be that among the most appropriate styles, some may appeal more to specific populations than others. For example, when creating a yoga intervention for stress reduction among adolescents, a vigorous style would be more appropriate than when creating such an intervention for elderly caregivers. For individuals with multiple health problems, simple and safe postures would likely be ideal. For persons who travel extensively, yoga requiring lots of props would not be desirable. Some styles have specific therapeutic principles for dealing with specific health conditions, while others use more general principles and believe that all yoga is health producing. Some special yoga programs have already been developed as a package and used in nonstudy settings.

When reviewing the recent yoga literature describing studies for back pain (*n* = 10) [32–41], other pain (*n* = 8) [42–49], cardiovascular disease (*n* = 13) [7–19], and cancer (*n* = 12) [20–31] (Table 1), studies from the West were more likely to describe yoga in terms of specific styles (68%) than those conducted in India (7%). Among those, Iyengar yoga was the most commonly studied (36%), while another 21% specifically mentioned hatha yoga.

The depth and degree of teacher training is a major consideration both for a study and for ensuring that any positive treatment results could be successfully disseminated. Yoga teacher training is highly variable across styles and the quality of instructors is similarly variable. A style with modest training standards might be less desirable than one with more intensive standards because, if the intervention was effective, it would be easier to disseminate to the general public with higher quality standards in place. Moreover, from a practical perspective, instructors versed in all traditions may not be available in all locations. Another consideration is whether the style of yoga in the study could be easily adapted for dissemination to patients if the study was successful. A rarely used style that could not be adapted by other teachers would not be of general interest. A style with an explicit emphasis on safety is critical for therapeutic use.

#### 2.1.2. Dose of Yoga

For studies, an appropriate dose must be established to optimize the potential value of a yoga protocol. Specifically, the critical questions are how much yoga needs to be practiced each week (frequency per week and minutes per session) and for how long (total number of weeks) in order to see therapeutic change and to promote longer term practice for chronic conditions, where individuals might need to practice yoga on a regular basis for an extended period of time to achieve optimal benefits. Inherent in this question is how much of the yoga should be practiced with an instructor and how much, if any, at home? Part of the answer to this question would deal with the quality and consistency of the practice. For example, is there some level of practice that is so substandard that no benefit would be expected? If so, the intervention should be long enough that the quality of the practice exceeds this threshold. How much exposure to an instructor is necessary to main adequate practice at home?

 Typically, yoga is taught in a class format in the West. However, therapeutically oriented yoga was originally customized to the needs of specific individuals and taught individually. Depending on the condition and the goals of the study, different formats could be used. But, it is likely that most clinical trials will use a class format.

In the absence of published studies on optimal dosing, new studies should review the literature for their condition (or related conditions).  Table 2 (and Table 4) illustrates the variations in the dose of yoga used in studies of pain, cancer, and cardiovascular disease. Intervention length was typically short (median of 8 weeks, with slightly longer for back pain). Most Western studies have included weekly or twice weekly classes, lasting between 60 and 90 minutes. Total class hours varied between a single class followed by a home intervention to a week long retreat (the latter in India), with a median of 16 hours. Most studies encouraged home practice. While some Asian studies have also been short in duration, they have included more classes per week and some were missing the information needed to determine the total exposure to yoga.

Because there is little information regarding the choice of dose, more work needs to be done to establish optimal treatment parameters, including exposure to yoga in a formal class, at home and whether other contact with instructors between formal classes should be facilitated. Because yoga is a skill, it may be appropriate for many people to practice at home once the safety issues are addressed and the components are learned properly.

#### 2.1.3. Components of the Yoga Intervention

Yoga as a spiritual discipline includes specific attitudes and behaviors (known as the “Yamas” and “Niyamas”), physical postures (known as “Asanas”), breathing exercises (known as “pranayama”), interiorization of the mind (Pratyahara), and progressively deeper states of concentration (Dharana) and meditation (Dhyana) leading to Oneness (Samadhi). In this context, all are considered valuable and practiced as a holistic package. Conceivably, all these elements could have therapeutic benefits for physical and mental health as well, so it is important to decide which of these (i.e., attitudes and behaviors, postures, breathing exercises, interiorization, and concentration/meditation) should be included in a yoga protocol for a particular condition. In order to promote the proper attitude for the safe practice of the intervention and to distinguish yoga from other therapies, the principles of yama and niyama should form a framework from which the instructor teaches the entire class. The components of a class might well differ from condition to condition. For example, therapy for back pain might focus on the practice of asana, with coordinated breathing, while therapy for hypertension might focus on meditation and therapy for complex conditions might include all of them.

A variety of techniques might be used in order to decide which elements should be included: consideration of the population (e.g., children may be unwilling to meditate), reviews of the treatment literature, consensus across a variety of more comprehensive styles using senior teachers, a formal Delphi process with senior instructors, and a consideration of yoga theory. However, formal studies testing the value of various techniques for specific populations would be desirable as well.

Table 3 indicates that most yoga classes include postures, breathing exercises, and relaxation in both Asia and the West. Meditation is more commonly taught in Asia than in the West. Other components, including philosophy, visualization, and Yoga Nidra, were less commonly mentioned. Conceivably, classes could contain more components than mentioned in the description.

#### 2.1.4. Specific Class Sequences

Yoga interventions are rarely described in detail in the biomedical literature. In some extant reports, there are apparently standardized sequences developed, while in others instructors select from a menu of asanas and/or pranayamas. Standardized sequences are more reproducible, but raise the question of whether multiple sequences would be appropriate for a study? If so, should the sequences be progressively more difficult (appropriate, for example, for rehabilitation from injury) or simply different but of comparable difficulty to keep the participants interested (e.g., for stress reduction)? In addition, would a prespecified sequence unduly constrains instructors from providing the best possible protocol, given the variability in abilities that might exist within different groups of participants?

When specifying sequences in detail, there is a need to consider variability due to physical limitations of the condition (e.g., not all back pain patients will be equally impaired), the presence of common comorbidities for some individuals (e.g., neck pain may be more common among back pain patients), and other important contextual factors (e.g., fitness). Finally, there is a question of whether sequences should be specifically designed for the condition of interest or should be focused on general health, reflecting the belief that yoga is of general salubrious benefits?

In general, the proposed sequences should be reviewed by expert nonstudy instructors familiar with this population. A reasonable exception might be when the class sequences are developed or approved by a very senior teacher in the style of interest or if the protocol is in widespread usage. After refinement of the protocol based on those comments, it is critical to pilot test the new intervention with some patients similar to those in the proposed study. The intention of this pilot test is to ensure that the teachers can deliver the intervention in the intended time frame, to discover any difficulties the participants might have in performing the postures and breathing exercises, or other components of the intervention. Such intervention development testing should be performed before any official studies are undertaken to ensure that the intervention can be delivered and practiced in a reproducible manner.

#### 2.1.5. Dealing with Modifications

If sequences are specifically detailed, some freedom is essential to adapt the postures to individual needs because of physical limitations or other conditions. In previous studies, our research group [40, 50, 51] has typically offered a clear alternative if a posture would be suitable for only a fraction of the participants. We have also provided the most common modifications (e.g., a chair variation).

#### 2.1.6. Instructors

Deciding on the qualifications of the instructors is clearly critical for the outcome of the study. Because yoga is not a licensed profession, it can be difficult to assure competency. From that perspective, establishing the minimum level of training and experience is critical but selection of instructors after having observed them teaching is also recommended. Even with well-trained and experienced instructors, it is important to train them on the protocol. For protocols that are intricate or challenging in any way, detailed training with practice teaching to promote consistency is recommended.

For some conditions and settings, it could be appropriate to require additional qualifications apart from yoga training (e.g., certification as a nurse or a physical therapist). Instructors should have a personal yoga practice that includes all elements of the yoga protocol.

#### 2.1.7. Home Practice

If home practice is believed to be an important part of the intervention, then aids to facilitate practice are recommended. These may include a home practice book with a description of the practice, a CD or DVD or internet materials to be used on the computer or a mobile phone. The options for aids to home practice are likely to increase in the future. A realistic perspective is necessary on the appropriate amount of home practice so that participants are actually likely to comply. In back pain studies, 20 to 30 minutes of home practice is recommended. If informal practice is desired (e.g., body awareness in daily activity), aids to facilitate this are recommended as well.

#### 2.1.8. Measurement of Intervention Fidelity over Time

Intervention fidelity, or the concept that core features of the intervention should be delivered as intended, is an area that has received increasing attention among behavioral medicine researchers [52]. Yet, little work in this area has been done for yoga. At the very least, a fraction of classes should be observed to ensure that instructors are delivering the interventions as designed. Checklists should be developed that specify the critical and minimum components of the intervention. For example, in a protocol with prespecified sequences, all elements of the sequence should be performed. Safety issues should be addressed by instructors for any postures or other elements that require special attention to reduce risk of injury. Other important aspects of instruction (e.g., the principle of truthfulness as applied to the practice of postures so that the practitioners are only doing what their body can actually do at that moment) should be included in the checklist as well. Evaluating these less tangible elements of yoga will be challenging, but critical, to ensure that the yoga actually reflects enough of the Indian roots that it can be recognized by seasoned practitioners as reflecting a practice designed to enhance awareness rather than simply exercise or mechanical breathing exercises.

## 3. Discussion

In this paper, a systematic yet flexible framework for developing treatment protocols for yoga trials is recommended. The proposed approach starts from the perspective of yoga as it is currently practiced (with variations in styles and components of the intervention) and recommends adaptations to the needs of each study.

We currently lack the necessary empirical data for establishing robust treatment protocols of yoga. Therefore, a number of alternative methods were suggested for assisting in this process, including consideration of yoga theory, literature reviews, consensus among senior teachers, the use of a formal Delphi process, and practical considerations. All of these methods rely to some degree on opinion rather than structured observations. As such, the biases of the participants cannot always be explicated and issues of loyalty to lineage or the potential of commercial profit from positive studies cannot always be excluded. Nonetheless, each of them also has certain strengths as well as weaknesses. For example, some aspects of yoga theory are classically based, transcend lineage, and define the discipline, such as universal compassion and attention to awareness. Other components, such as the theory of sequencing of postures, fit more clearly into specific styles of yoga. Reviews of the literature, even if comprehensive, can only include previously published studies. Because most studies do not report on the basis for their interventions, it is difficult to know whether these were carefully thought out and comprehensive in their development or relied on local instructors to come up with programs. Consensus among senior teachers is likely easier if they are selected from a single style or related styles. If the instructors are familiar with the condition, such an approach may be useful in coming up with protocols that have some chance for success. However, if the patient population is somehow different from those of the various instructors, their input may not be as valuable. The Delphi process can be cumbersome and time consuming and thus may not be attractive to the most knowledgeable instructors. In addition to appropriate attention to practical concerns, use of multiple methods is recommended at this point to minimize the weaknesses of each of them.

One caveat regarding the evaluation of yoga for medical concerns is the question of what precisely is yoga. Although developed as a holistic and multicomponent discipline, a lot of yoga practice in the West has been changed from its traditional roots. In fact, even many teachers lack a clear understanding of what makes yoga unique. Thus, it could be that many “therapeutic yoga interventions” are actually “therapeutic mind-body interventions inspired by yoga.” Nonetheless, they may offer useful benefits to persons with a variety of health conditions.

Much work needs to be done in developing further recommendations for treatment protocols. Paramount among those is development of a better understanding of optimal dosing. In addition to hours of class time, dosing is likely to include consideration of home practice and time allotted to specific elements of yoga (e.g., meditation, postures).

Currently, a variety of interventions fall under the rubric of yoga and their heterogeneity makes it difficult to summarize the evidence regarding yoga's therapeutic efficacy. However, developing a more scientifically rigorous understanding of some of the elements of yoga (postures, breathing exercises, relaxation, and meditation) and their physiological correlates might permit the development of future protocols based more directly on optimization of the underlying physiological parameters. By searching for common factors across styles (e.g., enhancing parasympathetic tone), such protocols might circumvent the need to look at the benefits of a variety of styles of yoga.

Because therapeutic yoga is traditionally individualized, it is conceivable that more standardized protocols will not provide optimum benefits for participants. There are several ways to test this hypothesis. One is to directly compare a standardized versus individualized protocol as separate arms of a clinical trial. Another is to try a standardized protocol and see if those who fail to improve on such a protocol do better with an individualized protocol. Such studies would be important in assisting both the research and the yoga community in understanding whether and by how much standardized research protocols are likely to underestimate the effects of various yoga interventions.

Other studies could investigate the value of other parts of the protocol, such as home practice in improving treatment outcomes. By empirical testing of these recommendations for developing yoga protocols, guidance on their relative value will be better refined. This, in turn, will allow more rigorous evaluation of the value of yoga for addressing chronic pain, various symptoms related to cancer and its treatment, cardiovascular health, and a variety of quality of life-related aspects of chronic disease.

Although empirical studies comparing various aspects of yoga protocols could well be valuable, researchers need to understand that the differences in effectiveness between such protocols are likely to be small. As a consequence, very large sample sizes would be needed to detect statistically significant differences. For example, in a study of yoga for back pain, 60% of those receiving yoga had improved by at least 50% at the end of treatment [34]. If we had compared this treatment with a more “optimal yoga” and expected 70% to improve this amount, the sample size would be over 450 persons per group. Another way to approach this challenge is to ask whether there are clinically important benefits between the two protocols. If only clinically unimportant differences were found when comparing various types of protocols (e.g., different styles of yoga) for a number of distinctly different conditions (e.g., symptoms such as pain or fatigue, mental health concerns, and physiologically based diseases), this would be reassuring for the public, who might not be able to find classes teaching the precise contents of the yoga intervention.

In the context of describing randomized trials, more detailed descriptions of yoga interventions will enhance the ability of researchers to judge the quality of a trial, to clearly understand what was done, and to replicate key clinical studies. By including additional elements for nonpharmacological clinical trials in the CONSORT statement [53], as now required by many journals for such trials, the routine reporting of yoga trials will be improved. For example, such elements as a description of the different components of the interventions, standardization of key features, and how the intervention was tailored to individuals will be routinely included. However, this does not unambiguously enumerate all principal domains that clearly describe yoga protocols and does not specify the level of detail that will be reported. Therefore, yoga researchers will benefit from creating a consensus document to specify the critical elements for fully reporting protocols of yoga interventions in clinical trials.

## Figures and Tables

**Table 1:** Styles of Yoga.

West [USA, Europe, Australia, Brazil, and Turkey]						
Yoga style	Back pain	Other pain	CVD	Cancer	Total	Percentage
Ashtanga Vinyasa			Cade 2010		1	4%
Classical				Ulger 2010	1	4%
Hatha	Galantino 2004		Pullen 2008	Moadal 2007	6	21%
			Pullen 2010			
	Saper 2009		van Montfrans 1990			
Iyengar	Jacobs 2004	Evans 2011 (IBS)		Banasik 2011	8	29%
		Evans 2011 (rheumatoid arthritis)				
	Williams 2005	Garfinkel 1998 (carpal tunnel)				
	Williams 2009	Kuttner 2007 (IBS)				
Iyengar-inspired				Culos-Reed 2006	2	7%
				Galantino 2012		
Multiple perspectives	Cox 2010				2	7%
	Tilbrook 2011					
Not stated		da Silva 2007 (fibromyalgia)	Cheema 2011		2	7%
Tibetan				Cohen 2004	1	4%
Viniyoga	Sherman 2005				2	7%
	Sherman 2011					
Yoga in daily life				Kovacic 2011	1	4%
Yoga of awareness (Kripalu influenced)		Carson 2011 (fibromyalgia)		Carson 2009	2	7%

Asia [India, Thailand]						

Comprehensive, no “stylistic details”	Tekur 2008	John 2007 (migraine)		Banerjee 2007	6	40%
				Raghavendra 2007		
				Rao 2008		
				Vadiraja 2009		
Kriya			Agte 2011		1	7%
Not stated		Telles 2009 (musculoskeletal hand discomfort)	Datey 1969		8	53%
			McCaffrey 2005			
			Murthy 2011			
			Pal 2011			
			Patel 1976			
			Selvamurthy 1998			
			Yogendra 2004			
				Sum	15	100%

*Specific styles of yoga or specific programs.

**Table:** (a) Dosing of yoga: summary of studies from the Western World*

West [USA, Europe, Australia, Brazil, and Turkey]				
	Back pain	Other pain	CVD	Cancer
Durations (weeks)				
*N*	9	6	5	8
Range	6–24 weeks	4–8 weeks	8–20 weeks	1–12 weeks
Median	12 weeks	7 weeks	9 weeks	7.5 weeks
Mode	12 weeks	8 weeks	8 weeks	none
Classes/week				
*N*	9	6	5	8
Range	1-2 classes/week	only 1 class-2/wk	1–3 classes/week	1–7 classes/week
Median	1 class/week	2 class/week	2 class/week	1 class/week
Mode	1 class/week	2 class/week	2 class/week	1 class/week
Length of single class (min.)				
*N*	9	6	5	7
Range	60–90 minutes	50–120 minutes	50–70 minutes	45–120 minutes
Median	75 minutes	60–90 minutes	60 minutes	75 minutes
Mode	75 minutes	90 minutes	60 minutes	90 minutes
Total hours of classes				
*N*	9	6	5	7
Range	12–72 hours	1–18 hours	8–26 hours	7–24 hours
Median	15 hours	16 hours	19 hours	16 hours
Mode	15 hours	18 hours	none	none
Home practice (any recommended)				
*N*	9	6	5	8
%Yes	100%	67%	60%	50%

*Further details are provided in Table 4(a).

**Table:** (b) Dosing of yoga: summary of studies from Asia*

Asia [India, Thailand]				
	Back pain	Other pain	CVD	Cancer
Durations (weeks)				
*N*	1	2	8	3
Range	1 week	8, 12 weeks	3–52 weeks	6 weeks
Median	1 week	na**	8 weeks	6 weeks
Mode	1 week	na	3 weeks	6 weeks
Classes/week				
*N*	1	2	5	2
Range	7 classes	5 classes	3–7 classes	3, 7 classes
Median	na	5 classes	4 classes	na
Mode	na	5 classes	3 classes	na
Length of single class (min.)				
*N*	1	2	6	4
Range	Retreat, 9 hrs/day	60 minutes	30–63 minutes	60–90 minutes
Median	na	60 minutes	30 minutes	60 minutes
Mode	na	60 minutes	30 minutes	60 minutes
Total hours of classes				
*N*	1	2	4	2
Range	56 hours	40, 60 hours	10.5–80 hours	18+, 25 hours
Median	na	na	13.5 hours	na
Mode	na	na	na	na
Home practice (any recommended)				
*N*	1	2	8	4
%Yes	0%	50%	25%	75%

*Further details are provided in Table 4(b).

**Not applicable.

**Table 3:** Components of Yoga.

West [USA, Europe, Australia, Brazil, and Turkey]						
Back pain	Other pain	CVD	Cancer	Total	Percentage
Postures	9 (Galantino 04, Jacobs 04, Williams 05, Sherman 05, Williams 09, Saper 09, Cox 10, Tilbrook 11, and Sherman 11)	6 (Carson 2011 (fibromyalgia), da Silva 2007 (fibromyalgia), Evans 2011 (IBS), Evans 2011 (rheumatoid arthritis), Garfinkel 1998 (carpal tunnel syndrome), and Kuttner 2007 (IBS))	5 (Cade 2010, Cheema 2011, Pullen 2008, Pullen 2010, and van Montfrans 1990)	7 (Banasik 2011, Carson 2009, Cohen 2004, Culos-Reed 2006, Galantino 2012, Moadal 2007, and Ulger 2010)	27	96%
Breathing	6 (Galantino 04, Sherman 05, Saper 09, Cox 10, Tilbrook 11, and Sherman 11)	3 (Carson 2011 (fibromyalgia), da Silva 2007 (fibromyalgia), and Kuttner 2007 (IBS))	5 (Cade 2010, Cheema 2011, Pullen 2008, Pullen 2010, and van Montfrans 1990)	7 (Carson 2009, Cohen 2004, Culos-Reed 2006, Galantino 2012, Kovacic 2011, Moadal 2007, and Ulger 2010)	21	75%
Relaxation	7 (Galantino 04, Jacobs 04, Sherman 05, Saper 09, Cox 10, Tilbrook 11, and Sherman 11)	2 (da Silva 2007 (fibromyalgia), Garfinkel 1998 (carpal tunnel syndrome))	4 (Cade 2010, Pullen 2008, Pullen 2010, and van Montfrans 1990)	4 (Culos-Reed 2006, Galantino 2012, Kovacic 2011, and Ulger 2010)	17	61%
Meditation	1 (Galantino 04)	1 (Carson 2011 (fibromyalgia))	4 (Cheema 2011, Pullen 2008, Pullen 2010, van Montfrans 1990)	3 (Carson 2009, Moadal 2007, Ulger 2010)	9	32%
Mental focus	1 (Tilbrook 11)		1 (Cade 2010)	1 (Kovacic 2011)	3	11%
Visualization				1 (Cohen 2004)	1	4%
Philosophy/ lifestyle	1 (Tilbrook 11)	2 (Carson 2011 (fibromyalgia), da Silva 2007 (fibromyalgia))			3	11%
Yoga Nidra				1 (Kovacic 2011)	1	4%

Asia [India, Thailand]						

Postures	1 (Tekur 2008)	2 (John 2007 (migraine), Telles 2009 (musculoskeletal hand discomfort))	6 (Agte 2011, McCaffrey 2005, Murthy 2011, Pal 2011, Selvamurthy 1998, Yogendra 2004)	4 (Banerjee 2007, Raghavendra 2007, Rao 2008, Vadiraja 2009)	13	87%
Breathing	1 (Tekur 2008)	2 (John 2007 (migraine), Telles 2009 (musculoskeletal hand discomfort))	5 (Agte 2011, McCaffrey 2005, Murthy 2011, Pal 2011, Patel 1976)	4 (Banerjee 2007, Raghavendra 2007, Rao 2008, Vadiraja 2009)	12	80%
Relaxation	1 (Tekur 2008)	2 (John 2007 (migraine), Telles 2009 (musculoskeletal hand discomfort))	6 (Datey 1969, McCaffrey 2005, Murthy 2011, Pal 2011, Patel 1976, Yogendra 2004)	3 (Raghavendra 2007, Rao 2008, Vadiraja 2009)	12	80%
Meditation	1 (Tekur 2008)	1 (John 2007 (migraine))	2 (Agte 2011, Patel 1976)	4 (Banerjee 2007, Raghavendra 2007, Rao 2008, Vadiraja 2009)	8	53%
Chanting	1 (Tekur 2008)		1 (Pal 2011)	3 (Banerjee 2007, Raghavendra 2007, and Vadiraja 2009)	4	27%
Mental Focus			2 (Datey 1969, Patel 1976)		2	13%
Visualization		1 (Telles 2009 (musculoskeletal hand discomfort))		3 (Banerjee 2007, Raghavendra 2007, and Rao 2008)	4	27%
Philosophy/ lifestyle	1 (Tekur 2008)		2 (Agte 2011, Yogendra 2004)		3	20%
Yoga Nidra				1 (Banerjee 2007)	1	7%

**Table:** (a)

West [U.S., Europe, Australia, Brazil, Turkey]						
	Back pain	Other pain	CVD	Cancer		
Durations (weeks)					Total	Percentage
1				1 (Kovacic 2011)	1	4%
4		1 (Kuttner 2007—IBS)		1 (Ulger 2010)	2	7%
6	1 (Galantino 04)	2 (Evans 2011—IBS, Evans 2011—rheumatoid arthritis)			3	11%
7				2 (Cohen 2004, Culos-Reed 2006)	2	7%
8		3 (Carson 2011—fibromyalgia, da Silva 2007—fibromyalgia, and Garfinkel 1998—carpal tunnel)	2 (Pullen 2008, van Montfrans 1990)	2 (Banasik 2011, Carson 2009)	7	25%
8–10			1 (Pullen 2010)		1	4%
10			1 (Cheema 2011)		1	4%
12	6 (Jacobs 04, Sherman 05, Saper 09, Cox 10, Tilbrook 11, and Sherman 11)			2 (Galantino 2012, Moadal 2007)	8	29%
16	1 (Williams 05)				1	4%
20			1 (Cade 2010)		1	4%
24	1 (Williams 09)				1	4%

Classes/week					Total	Percentage

1 hour total, then at home		1 (Kuttner 2007—IBS)			1	4%
1	5 (Sherman 05, Saper 09, Cox 10, Tilbrook 11, andSherman 11)	2 (Carson 2011—fibromyalgia, da Silva 2007—fibromyalgia)	1 (van Montfrans 1990)	4 (Carson 2009, Cohen 2004, Culos-Reed 2006, and Moadal 2007)	12	43%
2	4 (Galantino 04, Jacobs 04, Williams 05, Williams 09)	3 (Evans 2011—IBS, Evans 2011—rheumatoid arthritis, and Garfinkel 1998—carpal tunnel)	2 (Pullen 2008, Pullen 2010)	2 (Banasik 2011, Ulger 2010)	11	39%
2-3			1 (Cade 2010)		1	4%
3			1 (Cheema 2011)		1	4%
7				1 (Kovacic 2011)	1	4%
Varies				1 (Galantino 2012)	1	4%

Length of single class (min.)					Total	Percentage

45				1 (Kovacic 2011)	1	4%
50		1 (da Silva 2007—fibromyalgia)	1 (Cheema 2011)		2	7%
60	1 (Galantino 04)	1 (Kuttner 2007—IBS)	3 (Cade 2010, Pullen 2010, van Montfrans 1990)	1 (Ulger 2010)	6	21%
60–90		1 (Garfinkel 1998—carpal tunnel)			1	4%
70			1 (Pullen 2008)		1	4%
71				1 (Galantino 2012)	1	4%
75	5 (Sherman 05, Saper 09, Cox 10, Tilbrook 11, and Sherman 11)			1 (Culos-Reed 2006)	6	21%
90	3 (Jacobs 04, Williams 05, and Williams 09)	2 (Evans 2011—IBS, Evans 2011—rheumatoid arthritis)		2 (Banasik 2011, Moadal 2007)	7	25%
120		1 (Carson 2011—fibromyalgia)		1 (Carson 2009)	2	7%
Not stated				1 (Cohen 2004)	1	4%

Total hours of classes					Total	Percentage

1		1 (Kuttner 2007—IBS)			1	4%
6.67		1 (da Silva 2007—fibromyalgia)			1	4%
7				1 (Kovacic 2011)	1	4%
8			1 (van Montfrans 1990)	1 (Ulger 2010)	2	7%
8.75				1 (Culos-Reed 2006)	1	4%
12	1 (Galantino 04)				1	4%
15	5 (Sherman 05, Saper 09, Cox 10, Tilbrook 11, and Sherman 11)				5	18%
16		1 (Carson 2011—fibromyalgia)	1 (Pullen 2010)	1 (Carson 2009)	3	11%
16–18		1 (Garfinkel 1998—carpal tunnel)			1	4%
18		2 (Evans 2011—IBS, Evans 2011—rheumatoid arthritis)		1 (Moadal 2007)	3	11%
18.67			1 (Pullen 2008)		1	4%
21.3				1 (Galantino 2012)	1	4%
24				1 (Banasik 2011)	1	4%
25			1 (Cheema 2011)		1	4%
26	1 (Jacobs 04)				1	4%
40–60			1 (Cade 2010)		1	4%
48	1 (Williams 05)				1	4%
72	1 (Williams 09)				1	4%
Not stated				1 (Cohen 2004)	1	4%

Home practice (any recommended)					Total	Percentage

Yes	9 (Galantino 04, Jacobs 04, Williams 05, Sherman 04, Williams 09, Saper 09, Cox 10, Tilbrook 11, and Sherman 11)	4 (Carson 2011—fibromyalgia, Evans 2011—IBS, Evans 2011—rheumatoid arthritis, and Kuttner 2007—IBS)	3 (Cade 2010, Pullen 2010, and van Montfrans 1990)	4 (Carson 2009, Cohen 2004, Galantino 2012, and Kovacic 2011)	20	71%
No		2 (da Silva 2007—fibromyalgia, Garfinkel 1998—carpal tunnel)	2 (Cheema 2011, Pullen 2008)	4 (Banasik 2011, Culos-Reed 2006, Moadal 2007, and Ulger 2010)	8	29%

**Table:** (b)

Asia [India, Thailand]						
	Back pain	Other pain	CVD	Cancer		
Durations (weeks)					Total	Percentage

1	1 (Tekur 2008)				1	7%
3			3 (Datey 1969, Murthy 2011, and Selvamurthy 1998)		3	20%
6				2 (Banerjee 2007, Vadiraja 2009)	2	13%
6+				1 (Rao 2008)	1	7%
8		1 (Telles 2009—musculoskeletal hand discomfort)	2 (Agte 2011, McCaffrey 2005)		3	20%
9			1 (Patel 1976)		1	7%
12		1 (John 2007—migraine)			1	7%
24			1 (Pal 2011)		1	7%
52 (1 year)			1 (Yogendra 2004)		1	7%
Varies				1 (Raghavendra 2007)	1	7%

Classes/week					Total	Percentage

3			2 (McCaffrey 2005, Patel 1976)	1 (Vadiraja 2009)	3	20%
5		2 (Telles 2009—musculoskeletal hand discomfort), John 2007 (migraine))	1 (Pal 2011)		3	20%
7	1 (Tekur 2008)		1 (Selvamurthy 1998)	1 (Raghavendra 2007)	3	20%
Varies			1 (Yogendra 2004)	1 (Rao 2008)	2	13%
Not stated			3 (Agte 2011, Datey 1969, and Murthy 2011)	1 (Banerjee 2007)	4	27%

Length of each class (min.)					Total	Percentage

30			4 (Datey 1969, Murthy 2011, Patel 1976, and Selvamurthy 1998)		4	27%
35–40			1 (Pal 2011)		1	7%
60		2 (John 2007—migraine, Telles 2009—musculoskeletal hand discomfort)		3 (Raghavendra 2007, Rao 2008, and Vadiraja 2009)	5	33%
63			1 (McCaffrey 2005)		1	7%
90				1 (Banerjee 2007)	1	7%
Not stated	1 (Tekur 2008) —retreat with 9 hours of yoga/day		2 (Agte 2011, Yogendra 2004)		3	20%

Total hours of classes					Total	Percentage

10.5			1 (Selvamurthy 1998)		1	7%
13.5			1 (Patel 1976)		1	7%
18				1 (Vadiraja 2009)	1	7%
18+				1 (Rao 2008)	1	7%
25.2			1 (McCaffrey 2005)		1	7%
40		1 (Telles 2009—musculoskeletal hand discomfort)			1	7%
56	1 (Tekur 2008)				1	7%
60		1 (John 2007—migraine)			1	7%
70–80			1 (Pal 2011)		1	7%
Not stated			4 (Agte 2011, Datey 1969, Murthy 2011, and Yogendra 2004)	2 (Banerjee 2007, Raghavendra 2007)	6	40%

Home practice					Total	Percentage

Yes		1 (John 2007—migraine)	2 (Agte 2011, Yogendra 2004)	3 (Raghavendra 2007, Rao 2008, and Vadiraja 2009)	6	40%
No	1 (Tekur 2008)	1 (Telles 2009—musculoskeletal hand discomfort)	6 (Datey 1969, McCaffrey 2005, Murthy 2011, Patel 1976, Pal 2011, and Selvamurthy 1998)	1 (Banerjee 2007)	9	60%
